# ﻿New species and new records of *Monohelea* Kieffer (Diptera, Ceratopogonidae) from Brazil

**DOI:** 10.3897/zookeys.1136.86680

**Published:** 2022-12-19

**Authors:** Maria Clara Alves Santarém, Erick Aragão Cardoso, Israel de Souza Pinto, Maria Luiza Felippe-Bauer

**Affiliations:** 1 Coleção de Ceratopogonidae, Laboratório de Diptera, Instituto Oswaldo Cruz, Av. Brasil 4365, CEP 21040-900, Rio de Janeiro, RJ, Brazil Instituto Oswaldo Cruz Rio de Janeiro Brazil; 2 Curso de Ciências Biológicas, Instituto Federal de Educação, Ciência e Tecnologia do Pará, Campus Itaituba, R. Universitário, CEP 68183-300, Itaituba, Pará, Brazil Instituto Federal de Educação Itaituba Brazil

**Keywords:** Aquatic, biodiversity, Neotropical, predaceous midges, taxonomy

## Abstract

Two new Brazilian species of *Monohelea* Kieffer are described and illustrated based on male specimens, *Monoheleacapixaba***sp. nov.** from Espírito Santo and *Monoheleacoimbrai***sp. nov.** from Rio de Janeiro. New records for *M.aguirrei* Tavares & Souza, *M.archibaldoi* Tavares & Souza and *M.maculipennis* (Coquillet) are given based on specimens from Espírito Santo (all three species) and Amapá (*M.maculipennis* only). All specimens are deposited in the Ceratopogonidae Collection of Fundação Oswaldo Cruz, Brazil.

## ﻿Introduction

The predaceous genus *Monohelea* Kieffer is distributed worldwide and includes 97 extant species (Borkent and Dominiak 2020; Borkent et al. 2022). Santarém and Felippe-Bauer (2021) recognized 25 species from the Neotropics, of which 16 are cited as present in Brazil. *Monohelea* is included in the tribe Ceratopogonini, the females of which are known to be predators of other small insects, mainly chironomids (Diptera, Chironomidae) (Wirth and Grogan 1988). Downes (1978) reported females of *Monohelea* preying on swarming males of chironomids in Ontario, Canada. According to Wirth and Grogan (1981), the larvae are most frequently found in sphagnum moss and other bog-like habitats. Only a few species of *Monohelea* are known as immatures. Glukhova (1971, 1977) described the larva and discussed the habitat of species from Russia. Elson-Harris (1990) provided information about larva and pupa of *Monohelea* from Australia. Wirth and Grogan (1981) and Borkent (2014) described two pupae of the genus belonging to species from the United States (Maryland and New York), reared from sphagnum bog. Knowledge concerning the habitat of immature stages of *Monohelea* is lacking in the Neotropical region. The study of material deposited in the Ceratopogonidae Collection of Fundação Oswaldo Cruz (FIOCRUZ/CCER) revealed the presence of two undescribed species from the Brazilian States of Espírito Santo and Rio de Janeiro and new records of *Monoheleaaguirrei* Tavares & Souza, *M.archibaldoi* Tavares & Souza for Espírito Santo and *M.maculipennis* (Coquillet) for Espírito Santo and Amapá.

With the addition of the new species and the new records, there are now 27 species of *Monohelea* known from the Neotropics, 18 from Brazil and 11 from the Brazilian Amazon region.

## ﻿Materials and methods

Adult specimens were mounted on microscope slides in phenol-Canada balsam after the method described by Wirth and Marston (1968). Diagnostic characters were microphotographed using a NIKON Eclipse E 200 microscope with digital camera MOTICAM 2300, 3.0 MP, USB 2.0, and the plates were prepared using GIMP Portable 2.6. The general terminology is that employed in the paper on Brazilian *Monohelea* by Felippe-Bauer et al. (2017). Terms of the wing follow the system of the Manual of Central American Diptera (Borkent et al. 2009). All measurements are in micrometers, except those of the wings, which are in millimeters. This research is registered at SisGen (National System for the Management of Genetic Heritage and Associated Traditional Knowledge) under the number ABBD939. All specimens were deposited in the Ceratopogonidae Collection of Fundação Oswaldo Cruz (FIOCRUZ/CCER) and have been given a specimen registration number.

### ﻿ Monohelea capixaba

Taxon classificationAnimaliaDipteraCeratopogonidae

Santarém & Felippe-Bauer sp. nov.

ACF44B67-F53D-5017-B313-66BE67A70783

https://zoobank.org/B4C144D5-2EF4-42CF-876D-8860EC777758

Figs 1 , 2

#### Holotype.

**Male**, on microscope slide, labeled “Holotype *Monoheleacapixaba* Santarém and Felippe-Bauer”, “São Luiz de Baixo, Pancas, Espírito Santo, BRASIL, 19° 12'34.43"S, 40°49’13.75"W, 14.XII.2010, CDC light trap, mata, Pinto, I.S. [leg.]” (CCER#3699).

#### Diagnosis.

**Male adult**: The only Neotropical species of *Monohelea* with legs yellowish, hind femur with basal brown band, mesal brown stripe and subapical ventral brown spot; parameres triangular, greatly expanded at single base, tapering distally, with apical portion simple and pointed apex. Female adult: unknown.

#### Description.

**Figure 1.**
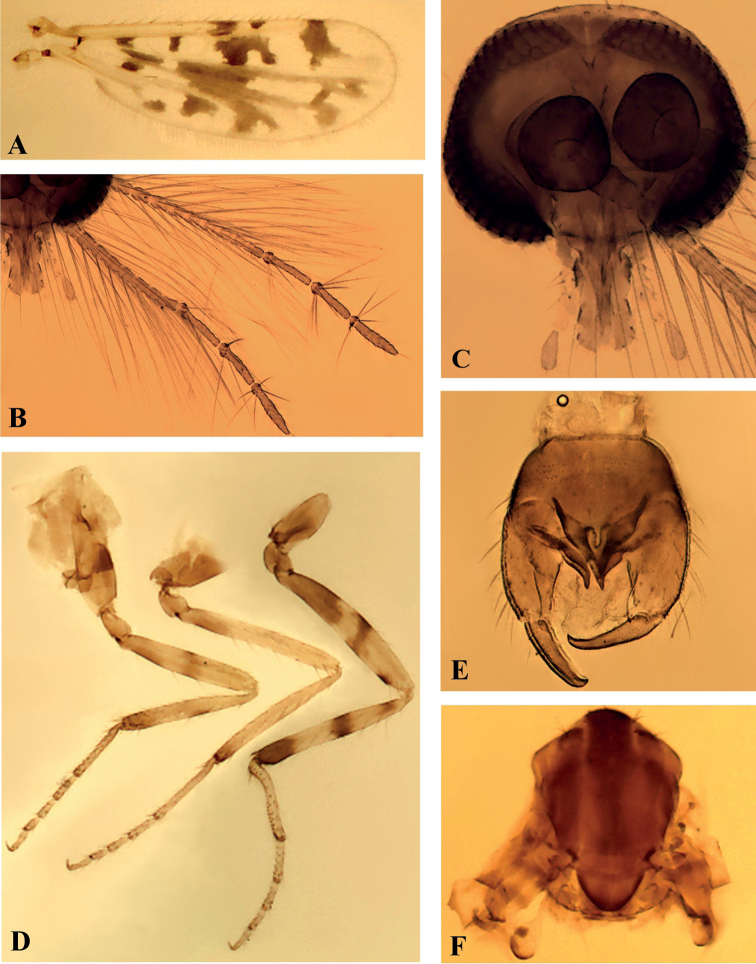
*Monoheleacapixaba* sp. nov., male **A** wing **B** antenna **C** head, anterior view **D** fore-, mid-, hind legs (left to right), lateral view **E** genitalia, ventral view **F** thorax, dorsal view.

**Male.** Head (Fig. 1C): eyes separated medially by a distance of 2 ommatidia. Antenna (Fig. 1B) brown; antennal ratio 0.95. Palpus pale brown, short; 3^rd^ segment with small, shallow sensory pit, 5^th^ segment darker, palpal ratio 1.29.

***Thorax*.** Scutum brown, two median pale strips (Fig. 1F). Legs (Fig. 1D) yellowish; coxae and trochanters brown; forefemur with basal brown band and mesal brown stripe, midfemur with basal brown band, hind femur with basal brown band, mesal brown stripe and subapical ventral brown spot; tibiae brown apically; hind tibia with subbasal spot, mesal stripe; tibiofemoral joints yellowish; hind tibial comb with 5 bristles. Tarsi pale; fore-, hind tarsomere 1 with one basal, one apical spine; midtarsomere 1 with 2 basal, 2 apical spines; apical spines of tarsomeres 2–4 of fore-, mid-, hind legs: 1-1-1, 2-2-2, 1-1-1; basal spines absent; fore-, mid-, hind tarsal ratios 2.14, 2.43, 1.87; claws small, paired, equal-sized, 0.43–0.48× as long as 5^th^ tarsomeres. Wing (Fig. 1A): macrotrichia present in wing margin; microtrichia absent; 2^nd^ radial cell nearly 2× longer than 1^st^; wing length 0.77 mm, width 0.30 mm; costal ratio 0.68. Halter pale, distal portion of knob darker.

**Figure 2.**
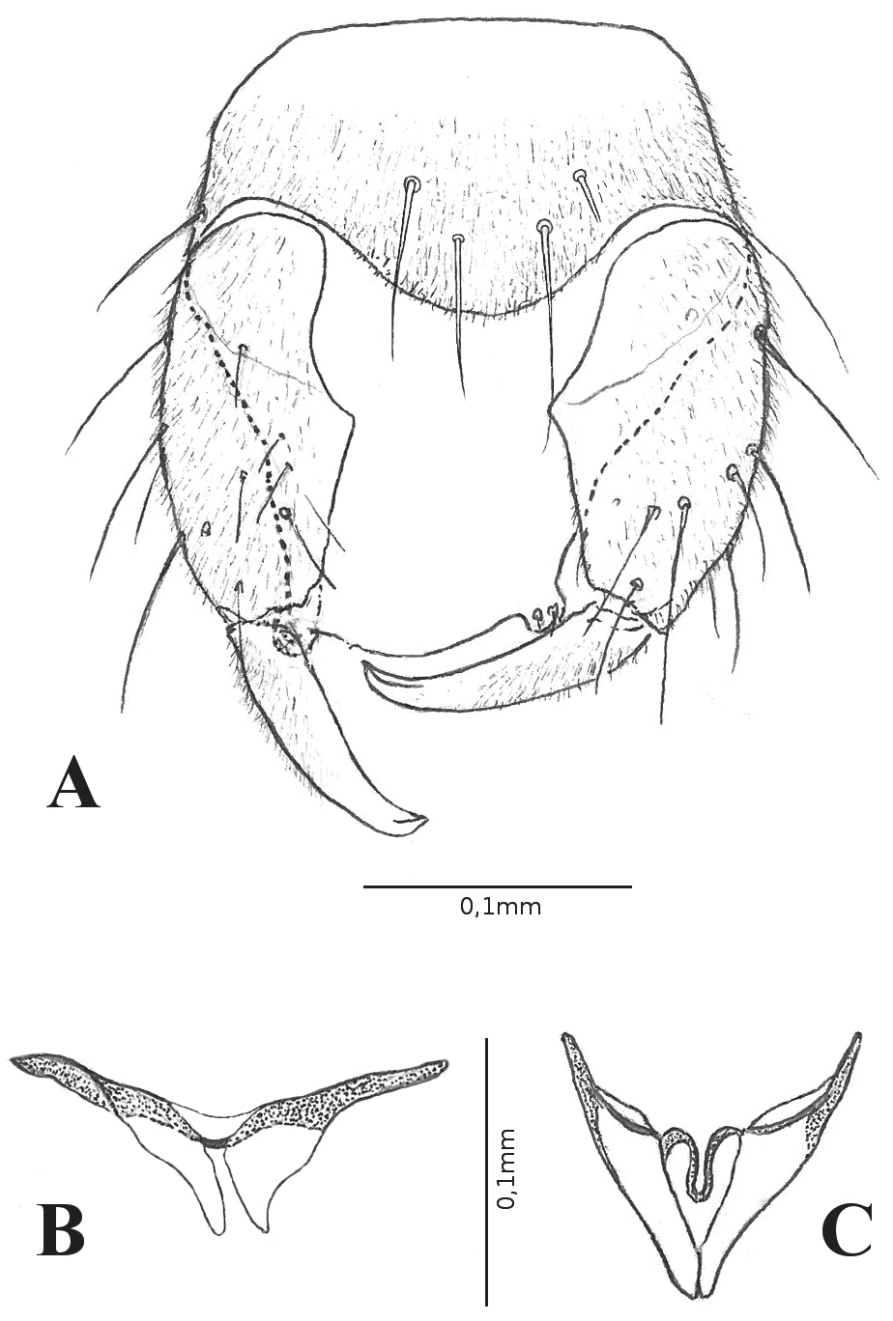
*Monoheleacapixaba* sp. nov., male **A** terminalia, ventral view **B** parameres, ventral view **C** aedeagus, ventral view.

***Abdomen*.** Pale brown. Genitalia brown (Figs 1E, 2A–C): sternite IX spiculate except on basal portion, posterior margin with a short, convex, median lobe with 4 long setae; tergite IX tapering, quadrate, with a pair of short apicolateral processes. Gonocoxite (Fig. 2A) moderately stout, nearly 2.06X longer than basal width, inner margin with mesal pointed protuberance; gonostylus (Fig. 2A) straight, tipped apex, 0.64 length of gonocoxite, basal 2/3 moderately pilose. Parameres (Fig. 2B) 0.91 length of aedeagus, triangular, greatly expanded at single base, tapering distally; apical portion simple, pointed apex. Aedeagus (Fig. 2C) triangular, composed of 2 pointed ventral plates; basal arms slightly expanded laterally.

**Female.** Unknown.

#### Distribution and bionomics.

This species is known only from forested areas in Espírito Santo State, Brazil.

#### Etymology.

This species name refers to the Portuguese gentilic name for the inhabitants of the Brazilian state of Espírito Santo, where the species occurs.

#### Taxonomic discussion.

The male of this species has yellowish legs with a pattern of brown patches and the brown base of the hind femur. This pattern is similar to the Brazilian Amazonian species *M.patauateua* Felippe-Bauer & Trindade. Also, this species keys to couplet 22 (male) of *M.mayeri* Ortíz and *M.hieroglyphica* Kieffer in the key to Neotropical *Monohelea* by Lane and Wirth (1964). It can be easily distinguished from these three species by the peculiar aspect of the triangular parameres with a single base, without a mesal process.

### ﻿ Monohelea coimbrai

Taxon classificationAnimaliaDipteraCeratopogonidae

Santarém & Felippe-Bauer sp. nov.

F8E62D2C-7F83-501E-8060-4CB73DE8B911

https://zoobank.org/B09403A-C4DC-44E1-84E2-D046A0B39BDB

Figs 3 , 4

#### Holotype.

**Male**, on microscope slide labeled “Holotype *Monoheleacoimbrai* Santarém and Felippe-Bauer”, “Rio Cascatinha, sessão de 2ª ordem (acima da represa) 1470 m, drift Caledônia, Nova Friburgo, Rio de Janeiro, BRASIL, 24.VIII.1995, Fittkau, UFRJ, IOC [leg.].” (CCER#3075).

#### Diagnosis.

**Male adult**: The only Neotropical species of *Monohelea* with legs pale, hind femur with large basal band slightly infuscated, mesal brown stripe, subapical ventral brown spot; gonostylus broad basally, tapering distally, deeply curved in distal 1/2; parameres stem swollen on proximal portion, curved, gradually tapering, internally directed, with small mesal pointed process, posteriorly directed arising from the swollen portion of the parameres. **Female adult**: unknown.

#### Description.

**Figure 3.**
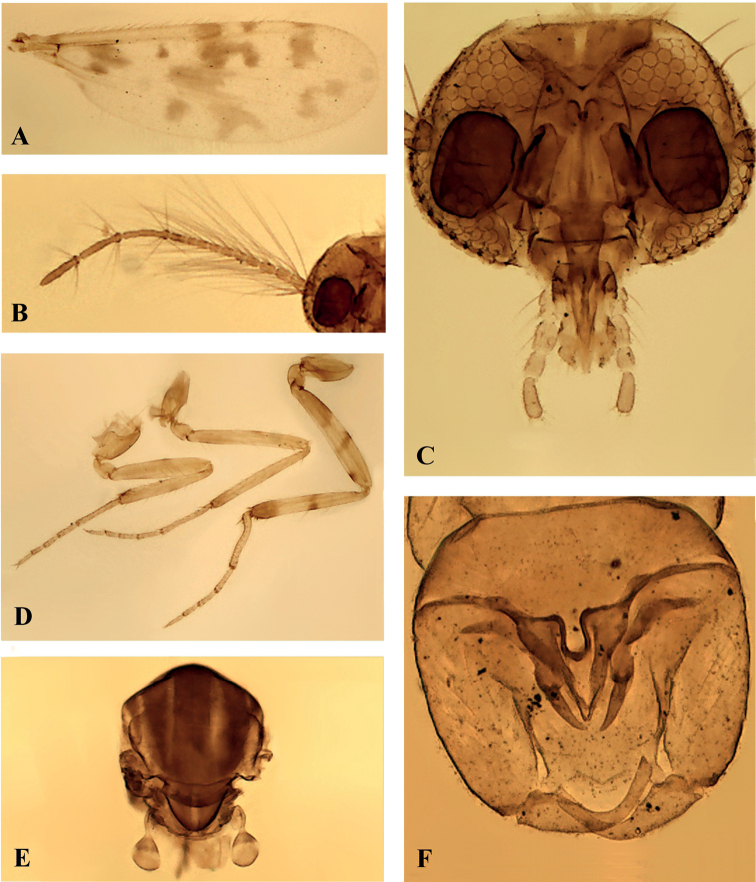
*Monoheleacoimbrai* sp. nov., male **A** wing **B** antenna **C** head, anterior view **D** fore-, mid-, hind legs (left to right), lateral view **E** thorax, dorsal view **F** genitalia, ventral view.

**Male.** Head (Fig. 3C): eyes separated medially by a distance of 2 ommatidia. Antenna (Fig. 3B) pale brown; antennal ratio 1.09. Palpus pale brown; 3^rd^ segment with small, shallow, sensory pit; palpal ratio 1.50.

***Thorax*.** Scutum (Fig. 3E) brown, pale brown laterally, two median pale strips. Legs (Fig. 3D) pale; coxae and trochanters pale; hind femur with large basal band slightly infuscated, mesal brown stripe and subapical ventral brown spot; hind tibia with mesal brown stripe, apical brown band; tibiofemoral joints yellowish; hind tibial comb with 7 bristles. Tarsi pale; fore-, hind tarsomere 1 with one basal, one apical spine; midtarsomere 1 with 2 basal, 2 apical spines; apical spines of tarsomeres 2–4 of fore-, mid-, hind legs: 1-1-1, 2-2-2, 1-1-1; basal spines absent; fore-, mid-, hind tarsal ratios 2.12, 2.34, 1.91; claws small, paired, equal-sized, 0.38–0.42× as long as 5^th^ tarsomeres. Wing (Fig. 3A): infuscated, macrotrichia restricted to costa; microtrichia absent; 2^nd^ radial cell nearly 2× longer than 1^st^; wing length 0.92 mm, width 0.35 mm; costal ratio 0.81. Halter pale brown.

**Figure 4.**
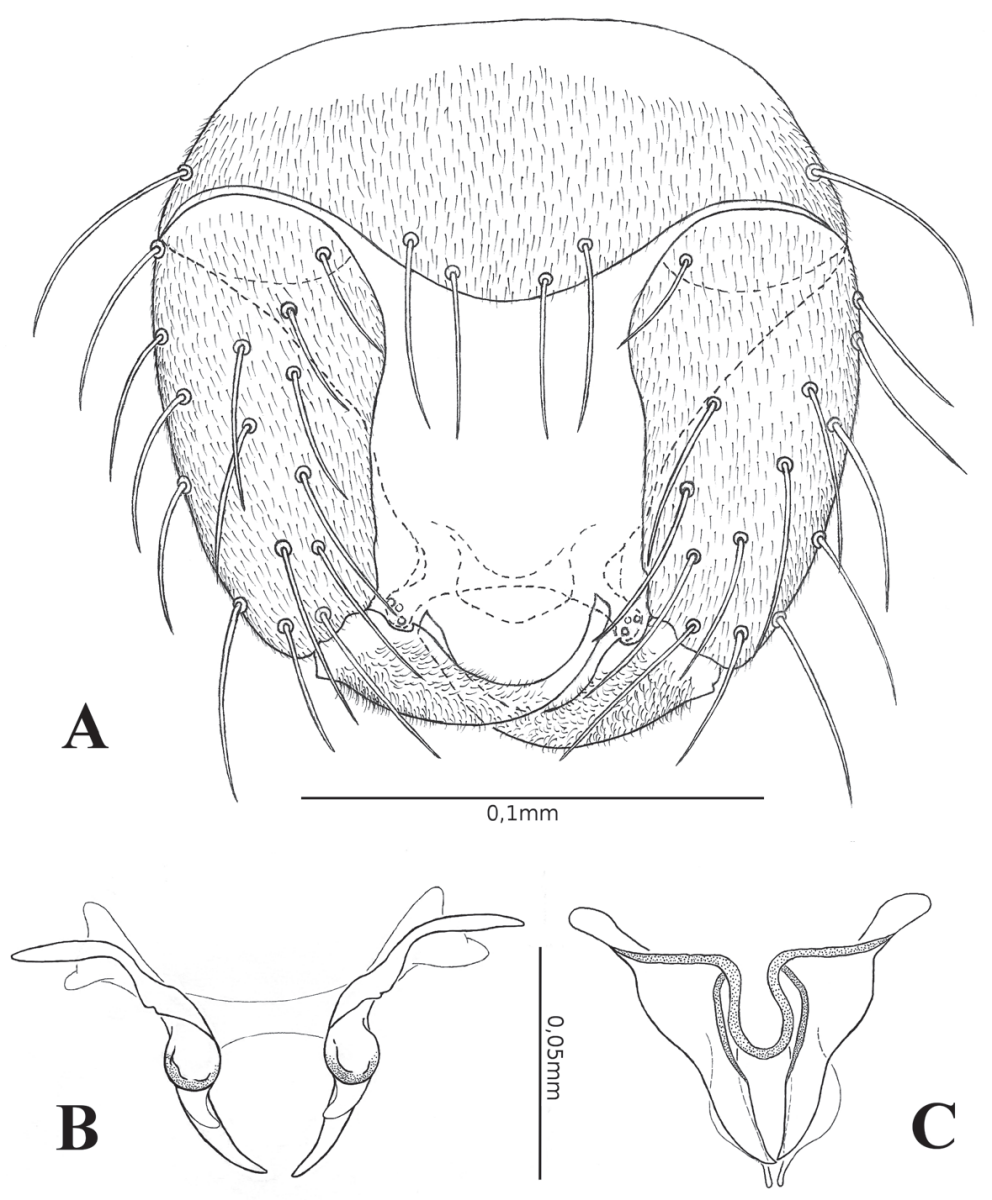
*Monoheleacoimbrai* sp. nov., male **A** terminalia, ventral view **B** parameres, ventral view **C** aedeagus, ventral view.

***Abdomen*.** Yellowish. Genitalia (Figs 3F, 4A–C) yellowish: sternite IX spiculate except on basal portion, posterior margin with a short, convex, median lobe with 4 long setae; tergite IX tapering, with a pair of short apicolateral processes, each with 3 setae. Gonocoxite (Fig. 4A) moderately stout, nearly 2× longer than basal width; gonostylus (Fig. 4A) broad basally, tapering distally, distal ½ deeply curved, apex blunt, 0.74 length of gonocoxite, moderately pilose on basal 1/2. Parameres (Fig. 4B) as long as aedeagus, fused at trilobed base, stem swollen, sclerotized on proximal portion, curved, gradually tapering, internally directed, with small inconspicuous mesal pointed process, tooth-shaped, posteriorly directed, arising from the swollen portion of the parameres. Aedeagus (Fig. 4C) triangular, composed of 2 pointed ventral plates, with slightly sclerotized dorsal structure, which arises in the middle way to aedeagus base and produced beyond the apices of ventral plates, ending as an apical projection; basal arms slender, broadly expanded laterally.

**Female.** Unknown

#### Distribution and bionomics.

This species is known only from Rio de Janeiro, Brazil. It has been found in forested areas near rivers, up to 1470 m above sea level.

#### Etymology.

This species is named in honor of Dr Adelmar Coimbra-Filho (1924–2016), a biologist and primatologist. He was an enthusiast of biodiversity conservation and acted on several strategies to protect the biodiversity of Atlantic Forest, where this species has been found.

#### Taxonomic discussion.

This species has pale legs with a pattern of brown patches and mesal processes in the parameres. It keys to couplet 19 in the key to Neotropical *Monohelea* by Lane and Wirth (1964), but it can be easily distinguished from *M.brasiliensis* Lane, *M.maculipennis* (Coquillet) and *M.poncai* Lane & Wirth by the presence of a unique, small inconspicuous mesal process of the parameres, tooth-shaped, posteriorly directed and arising from the swollen proximal portion. *Monoheleacoimbrai* sp. nov. has a single deeply curved gonostylus that is unique in the Brazilian species of this genus.

##### ﻿New records

### ﻿ Monohelea archibaldoi

Taxon classificationAnimaliaDipteraCeratopogonidae

Tavares & Souza, 1980

E60004FF-B6D0-5527-924B-DA8920A07762

Monohelea archibaldoi Tavares & Souza, 1980: 98 (male, female, Brazil - Rio de Janeiro); Wirth and Grogan 1988: 69 (type locality); Borkent and Wirth 1997: 101 (in catalog); Borkent and Spinelli 2000: 50 (in catalog); Felippe-Bauer and Oliveira 2001: 1111 (type specimens); Borkent and Spinelli 2007: 83 (in catalog); Borkent and Dominiak 2020: 165 (in catalog); Santarém and Felippe-Bauer 2021: 18 (in Brazilian catalog).

#### Distribution.

Brazil (Espírito Santo, Rio de Janeiro)

#### New records.

2 males, on microscope slides labeled “*Monoheleaarchibaldoi* Tavares & Souza, 1980”, “Comunidade de São Bento, Pancas, Espírito Santo, BRASIL, 19°13'44.0"S, 40°45'31.0"W, 06/II/2011, mata, CDC light trap, Pinto, I.S. [leg.]” (CCER#3700, CCER#3701).

### ﻿ Monohelea aguirrei

Taxon classificationAnimaliaDipteraCeratopogonidae

Tavares & Souza, 1980

D1CB92BD-E685-5326-9BB6-B30297228D13

Monohelea aguirrei Tavares & Souza, 1980: 97 (in part; male, Brazil - Rio de Janeiro); Wirth and Grogan 1988: 69 (type locality); Borkent and Wirth 1997: 101 (in catalog); Felippe-Bauer 1998: 223 (redescription); Borkent and Spinelli 2000: 50 (in catalog); Felippe-Bauer and Oliveira 2001: 1111 (type specimens); Borkent and Spinelli 2007: 83 (in catalog); Borkent and Dominiak 2020: 165 (in catalog); Santarém and Felippe-Bauer 2021: 18 (in Brazilian catalog).

#### Distribution.

Brazil (Espírito Santo, Rio de Janeiro, Santa Catarina) and Argentina (Corrientes, Buenos Aires Province).

#### New records.

1 male, 1 female, on microscope slides labeled “*Monoheleaaguirrei* Tavares & Souza, 1980”, “Palmital de Baixo, Pancas, Espírito Santo, BRASIL, 19°12'47.0"S, 40°47'20.0"W, 30/IX/2010, mata, CDC light trap, Pinto, I.S. [leg.]” (CCER#3702, CCER#3703); 1 male, same data except “Córrego Itauninhas, Mucurici, 18°04'11.8"S, 40°32'47.0"W, 02/IV/2010” (CCER#3704).

### ﻿ Monohelea maculipennis

Taxon classificationAnimaliaDipteraCeratopogonidae

(Coquillett, 1905)

C092ABE3-C9F2-50D3-BEE2-2615C52549C1

Ceratopogon maculipennis Coquillett, 1905: 64 (female, Fla.)
Monohelea maculipennis : Kieffer 1917: 312; Wirth 1953: 140 (redescr.; Mexico, Guatemala, Panama records; figs wing, female hind leg, male genitalia; discus.); Lane and Wirth 1964: 227 (distrib.; USA, Bahamas, Ecuador records; figs female hind leg, parameres; dimorphism); Wirth and Williams 1964: 308 (distrib.; fig. parameres; dimorphism); Wirth 1974: 41 (in catalog); Wirth and Grogan 1988: 69 (type locality); Borkent and Wirth 1997: 102 (in catalog); Felippe-Bauer 1998: 228 (Brazil records); Borkent and Spinelli 2000: 50 (in catalog); Borkent and Spinelli 2007: 83 (in catalog); Felippe-Bauer et al. 2017: 159 (Brazil - Pará record); Borkent and Dominiak 2020: 166 (in catalog); Santarém and Felippe-Bauer 2021: 18 (in Brazilian catalog).

#### Distribution.

USA (Florida, Louisiana), Mexico (Tamaulipas, Yucatan), Bahamas, Guatemala, Panama, Brazil (Amapá, Pará, Espírito Santo, Rio de Janeiro).

#### New records.

1 male on microscope slide labeled “*Monoheleamaculipennis* (Coquillet), 1905”, “Floresta Nacional do Rio Preto, Espírito Santo, BRASIL, 18°21'23.9"S, 39°50'41.4"W, 14/XII/2009, CDC light trap, Pinto, I.S. [leg.]” (CCER#3705); 2 males, same data except “Monumento Natural dos Pontões Capixabas, Palmital de Baixo, Pancas, 19°12'47.0"S, 40°47'20.0"W, 30/IX/2010, mata” (CCER#3706); “Córrego São Bento, Pancas, 19°13'50.0"S, 40°45'24.7"W, 18/X/2010, casa” (CCER#3707); 1 male on microscope slide labeled “*Monoheleamaculipennis* (Coquillet), 1905”, “Redenção, Amapá, BRASIL, 24/II/1964, Lacombe D. col.” (CCER#3123).

## Supplementary Material

XML Treatment for Monohelea capixaba

XML Treatment for Monohelea coimbrai

XML Treatment for Monohelea archibaldoi

XML Treatment for Monohelea aguirrei

XML Treatment for Monohelea maculipennis
